# Long-Term Exposure to Air Pollution Below Regulatory Standards and Cardiovascular Diseases Among US Medicare Beneficiaries: A Double Negative Control Approach

**DOI:** 10.21203/rs.3.rs-3530201/v1

**Published:** 2023-11-21

**Authors:** Yichen Wang, Danesh Yazdi Mahdieh, Yaguang Wei, Joel Schwartz

**Affiliations:** Harvard T.H. Chan School of Public Health; Stony Brook University; Harvard T.H. Chan School of Public Health; Harvard T.H. Chan School of Public Health

**Keywords:** Air Pollution, Double Negative Control, Stroke, Heart Failure, Atrial Fibrillation

## Abstract

Growing evidence suggests that long-term air pollution exposure is a risk factor for cardiovascular mortality and morbidity. However, few studies have investigated air pollution below current regulatory limits, and causal evidence is limited. We used a double negative control approach to examine the association between long-term exposure to air pollution at low concentrations and three major cardiovascular events among Medicare beneficiaries aged ≥ 65 years across the contiguous United States between 2000 and 2016. We derived ZIP code-level estimates of ambient fine particulate matter (PM_2.5_), nitrogen dioxide (NO_2_), and warm-season ozone (O_3_) from high-resolution spatiotemporal models. The outcomes of interest were hospitalizations for stroke, heart failure (HF), and atrial fibrillation and flutter (AF). The analyses were restricted to areas with consistently low pollutant levels on an annual basis (PM_2.5_ <10 μg/m^3^, NO_2_ < 45 or 40 ppb, warm-season O_3_ < 45 or 40 ppb). For each 1 μg/m^3^ increase in PM_2.5_, the hospitalization rates increased by 2.25% (95% confidence interval (CI): 1.96%, 2.54%) for stroke and 3.14% (95% CI: 2.80%, 3.94%) for HF. Each ppb increase in NO_2_ increased hospitalization rates for stroke, HF, and AF by 0.28% (95% CI: 0.25%, 0.31%), 0.56% (95% CI: 0.52%, 0.60%), and 0.45% (95% CI: 0.41%, 0.49%), respectively. For each ppb increase in warm-season O_3_, there was a 0.32% (95% CI: 0.21%, 0.44%) increase in hospitalization rate for stroke. The associations for NO_2_ and warm-season O_3_ became stronger under a more restrictive upper threshold. Using an approach robust to omitted confounders, we concluded that long-term exposure to low-level PM_2.5_, NO_2_, and warm-season O_3_ was associated with increased risks of cardiovascular diseases in the US elderly. Stricter national air quality standards should be considered.

## 1. Introduction

Long-term exposure to air pollution has been recognized as an important modifiable risk factor for cardiovascular diseases ^1,2^. An increasing number of epidemiological studies support positive associations between long-term air pollution and the occurrence of cardiovascular events, although specific cardiovascular outcomes have been less investigated relative to overall cardiovascular mortality and morbidity. Stroke, which is characterized by high incidence and mortality, is the second leading cause of death worldwide ^3^. Researchers have reported that long-term exposure to air pollution, particularly fine particulate matter with an aerodynamic diameter less than 2.5 μm (PM_2.5_), could be associated with an increased risk of hospitalization, incidence, and mortality due to stroke ^4^. Heart failure (HF) and atrial fibrillation (AF) are other two major cardiovascular diseases. They are important risk factors for stroke onset ^5^. Several studies demonstrated the adverse effect of long-term air pollution on the risk of HF ^6–8^ and AF ^9,10^, although these two endpoints have been understudied as primary outcomes of interest. Overall, the evidence for the hypothesized association, especially with HF and AF, remains scarce and inconsistent. In addition, with the predominant focus on PM_2.5_, the potential cardiovascular effects of long-term exposure to other major air pollutants such as nitrogen dioxide (NO_2_) and ozone (O_3_) have been under-examined, and the correlations between different pollutants have also been overlooked. To conclude, the potential causal relationships between multiple air pollutants and specific cardiovascular events need to be further elucidated.

Most of the existing studies linking long-term exposure to air pollution to cardiovascular events examined the entire range of exposure. The average pollution levels may differ substantially by region and therefore partially account for the geographical differences in the estimated associations. There is a dearth in our understanding of the health impacts of air pollution at concentrations below regulatory standards, which has important implications for air pollution regulations in regions such as the United States (US) where populations experience generally low air pollutant exposures. Previous studies found the shape of the exposure-response curves for long-term PM_2.5_ and all-cause and cardiovascular mortality to be curvilinear with no evidence of a threshold ^11,12^. According to several studies of large cohorts in the US ^9,13,14^ and Europe ^15,16^, the risk of cardiovascular diseases could persist and even become stronger at lower exposure levels below the annual limit values set by the US Environmental Protection Agency (EPA) and European Union (EU). The suggested higher incremental risk in relation to a lower air pollutant level raises the question of whether the national and international air quality guidelines are protective enough. Further research specifically at lower concentrations can help elucidate this.

Furthermore, many observational studies fail to utilize causal modeling methods to identify confounding and eliminate non-causal associations, therefore, they may yield estimates that lack validity to some extent. Propensity scores are the most widely adopted approach to simulate counterfactuals in randomized trials by balancing measured covariates between the exposed group and unexposed group or across different levels of continuous exposure in air pollution research. However, this method is weakened by its stringent requirement for precisely specified regression of exposure on measured covariates and its inability to control for unmeasured covariates. Negative controls have been suggested as a useful tool to enhance causal inference independently of covariate distributions and to tackle unmeasured confounding bias ^17^. The negative exposure control is a variable known not to be causally related to the outcome of interest, while the negative outcome control is a variable known not to be caused by the exposure of interest. Both of them may share a common confounding mechanism with the exposure and outcome ^18^. Therefore, they can serve as instruments for reducing bias by unmeasured confounders. In prior air pollution and health studies, researchers have used future air pollution as a negative control exposure ^19–22^, or a negative outcome due to causes other than primary exposure as a negative control outcome ^23,24^. More recently, double negative control adjustment has been employed to strengthen causal inference in studies examining short- and long-term effects of air pollution ^25–27^.

To address the research gaps, the present study used a double negative control approach to analyze the relationships between long-term exposure to PM_2.5_, NO_2_, and warm-season O_3_ at low concentrations with risk of hospitalizations for three major cardiovascular diseases (stroke, HF, and AF) in the Medicare population aged ≥ 65 years across the contiguous US from 2000 to 2016. We focused on the areas where populations were consistently exposed to low pollutant concentration levels (PM_2.5_<10 μg/m^3^, NO <40 or 20 ppb, warm-season O_3_ < 45 or 40 ppb). Furthermore, we conducted stratified analyses to investigate potential susceptible demographic subpopulations.

## 2. Methods

### 2.1. Study Population and Outcome Assessment

We used data from a national cohort of fee-for-service (FFS) Medicare beneficiaries aged 65 years and older across the contiguous US from January 1st, 2000 to December 31st, 2016. The beneficiaries were followed up from January 1st of the year after their Medicare enrollment until the development of the outcome of interest, death, censoring, or the end of the follow-up time. In this study, we restricted the analyses to the individuals who were consistently exposed to low-level annual air pollution for the entire period (2000–2016) with certain thresholds (PM_2.5_<10 μg/m^3^, NO_2_ < 40 or 20 ppb, warm-season O_3_ < 45 or 40 ppb). Therefore, three datasets were created for each pollutant according to its specified threshold. We further restricted the datasets to ZIP code areas with at least 100 beneficiaries.

Beneficiary records were provided by the Medicare denominator file from the Centers for Medicare and Medicaid Services, which contained information on age, self-reported sex, self-reported race, Medicaid eligibility, date of death, and residential ZIP code for each beneficiary. Information on age, Medicaid eligibility, and residential ZIP code are updated each year. We obtained the hospital discharge claims of Medicare enrollees from the Medicare Provider Analysis and Review (MEDPAR) file. The International Classification of Diseases (ICD) codes were used to identify the primary discharge diagnosis for each of our three cardiovascular outcomes of interest: stroke (ICD-9 codes: 430–438, ICD-10 codes: I60-I69), heart failure (ICD-9 code: 428, ICD-10 code: I50; hereafter referred to as HF), and atrial fibrillation and flutter (ICD-9 code: 427.3, ICD-10 code: I48; hereafter referred to as AF). For each cardiovascular outcome, we computed the ZIP code-level annual counts based on the beneficiaries’ residential addresses.

This study was approved by the institutional review board at Harvard T. H. Chan School of Public Health. It was exempt from informed consent requirements as a study of previously collected administrative data.

### 2.2. Exposure Assessment

We obtained the daily concentrations of ambient PM_2.5_, NO_2_, and O_3_ at 1 km×1 km spatial resolution across the contiguous US from three ensemble prediction models that combined multiple machine learning algorithms ^28–30^. The exposure models incorporated meteorological variables, chemical transport model simulations, land-use features, and satellite remote sensing data. They were well validated using 10-fold cross-validation. We aggregated the daily predictions of PM_2.5_ and NO_2_ to annual averages. For long-term O_3_, we calculated its warm-season levels based on the daily predictions from April 1st through September 30th, since the health impacts of O_3_ are suggested to be more observable during warm seasons compared to throughout the year ^13,31,32^. We then computed the ZIP code-level exposures by averaging the 1 km×1 km grid cell predictions whose centroids were within the boundary of ZIP code polygons or assigning the nearest grid cell predictions for the ZIP codes that do not have polygon representations. Annual average exposures were then linked to Medicare beneficiaries based on their residential ZIP codes for each calendar year over the study period.

For each exposure, we limited our dataset to the ZIP code areas where the populations were always exposed to low-concentration air pollution below thresholds we set over the study period of 2000–2016. We chose 10 μg/m^3^ as the threshold for annual average PM_2.5_ concentration, because this value has been proposed by the US EPA’s Clean Air Scientific Advisory Committee to substitute the current National Ambient Air Quality Standards (NAAQS) of 12 μg/m^3 33^. For NO_2_, we chose an annual limit of 40 ppb and an even lower limit of 20 ppb for our analysis, well below the NAAQS standard of 53 ppb, as the annual NO_2_ concentrations in the US rarely exceeded this standard. Although there is no formal annual regulatory standard for long-term O_3_, we selected 45 and 40 ppb as the threshold values to define low-level O_3_, which has been chosen as a plausible pollution target in previous studies to evaluate its effectiveness in reducing health risk ^34,35^.

### 2.3. Covariates

We considered a variety of SES covariates at the ZIP code tabulation-area (ZCTA) level, including percent of the population self-reporting as Black, percent of the population self-reporting as Hispanic, percent of the population ≥ 65 years of age living in poverty, population density, percent of the population ≥ 65 years of age who had not graduated from high school, median home value, median household income, and percent of owner-occupied housing unit. These data were obtained from the U.S. Census Bureau 2000 and 2010 Census Summary File 3 and the American Community Survey from 2011 through 2016. To account for long-term smoking behaviors, we included lung cancer hospitalization rates as a surrogate measure for each ZIP code from the MEDPAR file. We also accessed county-level data on the yearly percentage of residents who ever smoked and mean body mass index (BMI) from the Centers for Disease Control and Prevention (CDC) Behavioral Risk Factor Surveillance System (BRFSS) ^36^. These county-level lifestyle data were assigned to ZIP codes. Additionally, from the Dartmouth Atlas of Health Data ^37^, we obtained several access-to-care covariates in each hospital service area, and further assigned them to ZIP codes: proportion of Medicare beneficiaries with at least 1 hemoglobinA1c test per year, proportion of diabetic beneficiaries who had a lipid panel test in a year, proportion of beneficiaries who had an eye examination in a year, proportion of beneficiaries with at least 1 ambulatory doctor visits in a year, and proportion of female beneficiaries who had a mammogram during a 2-year period. We also calculated the distance from the centroid of each ZIP code to the nearest hospital, a proxy for healthcare accessibility, using data on hospital locations derived from an ESRI dataset ^38^. Given that seasonal meteorological conditions have been known to impact cardiovascular health ^39,40^, we assessed the average temperature and relative humidity (RH) during the summer (June-August) and the winter (December-February) for each ZIP code and each year based on the 4 km Gridded Surface Meteorological (gridMET) dataset ^41^. Missing values for all area-level risk factors were filled in using linear interpolation and extrapolation. Any other missingness accounting for < 1% of the observations was assumed to be random and was excluded from our analyses.

### 2.4. Statistical analysis

In this study, we analyzed the association between long-term exposure to low-level air pollution and hospitalization rate of major cardiovascular diseases among the US Medicare population. As aforementioned, the analysis was restricted to the low pollution ZIP code areas with at least 100 Medicare beneficiaries. We used a double negative control strategy, which has been recommended to address unmeasured confounding and other bias issues in observational settings ^17,42^, to enhance the causal evidence of a potential relationship. The detailed descriptions of this double negative control approach can be found elsewhere ^27^. A summary of the principles is given below. First, we consider a quasi-Poisson regression model to obtain the unbiased association between the exposure (A) and the outcome (Y), adjusting for unmeasured confounders (U):

1 $$ln[E(Y)]=\beta_{Y0}+\beta_{YA}A+\beta_{YU}U$$

The negative exposure control (Z) and negative outcome control (W) are designed to capture confounding bias introduced by U. In this study, we chose the exposure to air pollution in the year after cause-specific hospitalizations as Z. It cannot lead to the hospitalization outcome in the concurrent year, however, it could be influenced by unmeasured or measured confounders that are correlated with air pollution level in the year of the hospitalization outcome. Similarly, we defined the count of cause-specific hospitalizations in the year before exposure as W, as it is by no means affected by the exposure in the concurrent year but may be correlated to omitted confounders. Given the hypothesized correlations of U with A and Z, and non-causality between A and W, the formulas (2) and (3) can be derived:

2 $$E(U)=\beta_{U0}+\beta_{UA}A+\beta_{Uz}Z$$

3 $$ln[E(W)]=\beta_{WY0}+\beta_{WU}U$$

If we substitute U with its expected value regressed on A and Z from the formula (2), the formula (1) can be interpreted into:

4 $$ln[E(Y)]=\left(\beta_{Y0}+\beta_{YU}\beta_{U0}\right)+\left(\beta_{YA}+\beta_{YU}\beta_{UA}\right)A+\beta_{YU}\beta_{Uz}Z$$

where $\beta_{YU}\beta_{UA}$ is exactly equal to the bias due to unmeasured confounders. Thus, if the equation $\beta_{Uz}=\beta_{UA}$ holds, the subtraction between the coefficient of A and the coefficient of Z will yield a causal effect of A on Y.

If we substitute U with its expected value again in the formula (3), W as a surrogate for U can be predicted by A and Z based on:

5 $$ln[E(W)]=\left(\beta_{WU}\beta_{U0}\right)+\beta_{WU}\beta_{UA}A+\beta_{WU}\beta_{Uz}Z$$

Alternatively, assuming the linear correlations of U with A and Z, which renders the formulas (2) and (5) valid, we can mitigate the confounding effect of U by including the predicted W in the outcome regression model.

In the models, we adjusted for a variety of area-level risk factors for cardiovascular diseases selected prior, including SES, behavioral, and meteorological covariates which are described in the covariates section, to relax our assumptions and to reduce any uneliminated confounding bias. We also included the admission year as a categorical indicator in the models to control for the time trends of omitted confounders that might drive an association. We analyzed the effect of each air pollutant separately using both a single-pollutant model and a three-pollutant model. As a secondary analysis, we repeated the main analyses using generalized linear models (GLM) without the negative controls.

We examined the potential effect measure modification by individual demographic characteristics, namely, age (65–74 years, 75–84 years, 85 + years), sex (male or female), race (White or Black), and Medicaid eligibility (yes or no), using stratified analyses. We conducted pairwise comparisons of coefficients within the strata of each factor to detect any statistically significant differences, assuming the difference between the coefficients to follow a normal distribution with a mean of zero and a variance of the sum of the strata variances.

In the above analyses, we reported the effect as the percent change in hospitalization rate and its 95% confidence intervals (CIs) for each cardiovascular outcome per μg/m^3^ increase in annual exposure to PM_2.5_ and per ppb increase in annual exposure to NO_2_ and O_3_. All analyses were performed using R software version 4.2.3 on the Research Computing Environment as part of Research Computer at Harvard University Faculty of Arts and Sciences. A two-sided *P* value < 0.05 was considered statistically significant.

## 3. Results

Table 1 shows the summary statistics of ZIP code-level air pollution and covariates in the low-pollution areas from 2000 through 2016. In low PM_2.5_ areas, the annual average concentrations of PM_2.5_, NO_2_, and warm-season O_3_ were 5.9 ± 1.8 μg/m^3^, 12.8 ± 7.6 ppb, and 44.6 ± 7.3 ppb, respectively. In the areas with either NO_2_ or O_3_ deemed low in our analyses, the mean annual PM_2.5_ concentration was higher and closer to the typical range. The Pearson correlation coefficients (r) for three air pollutants are presented in **Supplementary Table 1**. We observed a moderate-to-low positive correlation between annual PM_2.5_ and NO_2_ in low NO_2_ areas (r = 0.38 and 0.23 at the thresholds of 40 and 20 ppb, respectively) and in low PM_2.5_ areas (r = 0.17). In contrast, there was a strong correlation between annual PM_2.5_ and NO_2_ in areas with low warm-season O_3_, with r values of 0.66 and 0.64 at the thresholds of 45 and 40 ppb, respectively. Warm-season O_3_ exhibited a moderate-to-low correlation with both annual PM_2.5_ and NO_2_ in areas with low levels of PM_2.5_ and NO_2_, while in areas with lower warm-season O_3_, the correlations were negligible.

**Supplementary Table 2** presents the total number of hospitalizations and the annual rate for stroke, HF, and AF in the low pollution areas during the study period. The annual hospitalization rate for stroke, HF, and AF among the Medicare participants were 0.97%, 0.96%, and 0.46%, respectively, in low PM_2.5_ areas where low NO_2_ and O_3_ exposures concurrently occurred. The corresponding hospitalization rates were similar in low O_3_ areas with both thresholds. However, the hospitalization rates were higher in low NO_2_ areas where people experienced more normal PM_2.5_ exposures. Nevertheless, the pattern of the hospitalization rates for each cardiovascular outcome within demographic groups was generally similar across all the defined low pollution areas. Overall, we observed higher annual hospitalization rates for stroke and HF among those aged 85 years and older and eligible for Medicaid. However, there were some inconsistencies in the pattern by sex and race across specific outcomes. While the annual hospitalization rate for stroke and HF was higher in males and black individuals, more AF hospitalizations occurred in females and white individuals.

**Figure 1:**
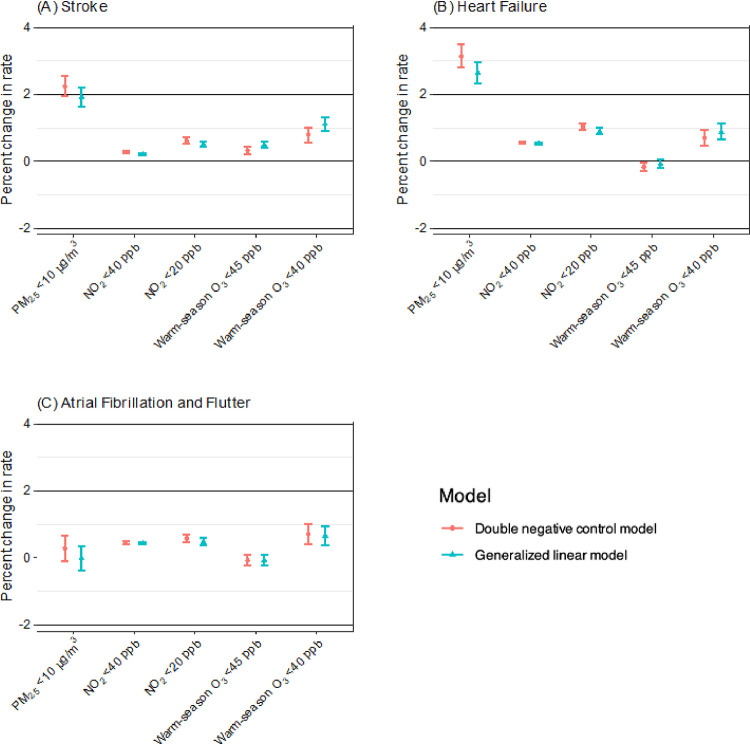
Percent change in hospitalization rate for stroke, HF, and AF associated with 1-μg/m^3^ increase in long-term exposure to PM_2.5_ and 1 ppb increase in long-term exposure to NO_2_ and warm-season O_3_ at low concentrations using double negative control models and generalized linear models adjusted for co-pollutants.

Figure 1 shows the associations of long-term exposures to PM_2.5_, NO_2_, and O_3_ at low concentrations with the rates of hospitalizations for stroke, HF, and AF as determined from three-pollutant double negative control models and GLM. The estimated associations from single-pollutant models are illustrated in **Supplementary Fig. 1**. Overall, the adjustments for co-pollutants resulted in stronger estimates for PM_2.5_, while those for NO_2_ and warm-season O_3_ remained similar. When examining the associations between PM_2.5_ and all three outcomes, we found that the GLM yielded estimates that were modestly comparable but lower than those derived from the double negative control models. While both modeling approaches produced relatively similar estimates for the associations of NO_2_ and warm-season O_3_ with AF, there were slight differences in the estimates for stroke and HF. All the numeric results of the overall analyses can be found in **Supplementary Table 3**.

In this study, we focused on the results adjusted for co-pollutants using double negative control adjustment. For long-term PM_2.5_ exposure below 10 μg/m^3^, we found that each 1-μg/m^3^ increase in annual PM_2.5_ concentration was associated with the percent increases of 2.25% (95% CI: 1.96%, 2.54%) and 3.14% (95% CI: 2.80%, 3.49%) in the hospitalization rates for stroke and HF, respectively. However, the association with AF was merely marginally significant with an estimate of 0.28% (95% CI: −0.10%, 0.67%). We observed adverse effects on all three outcomes associated with long-term exposure to NO_2_ at concentrations below 40 and 20 ppb. Specifically, for each 1-ppb increase in annual NO_2_ below 40 ppb, we estimated the percent increases in the hospitalization rates for stroke, HF, and AF to be 0.28% (95% CI: 0.25%, 0.31%), 0.56% (95% CI: 0.52%, 0.60%), and 0.45% (95% CI: 0.41%, 0.49%), respectively. At a lower threshold of 20 ppb, these estimates increased to 0.62% (95% CI: 0.54%, 0.71%), 1.04% (95% CI: 0.94%, 1.14%), and 0.59% (95% CI: 0.47%, 0.70%), respectively. Regarding the health effects of long-term exposure to warm-season O_3_ below 45 ppb, we found an adverse effect on stroke only with a 0.32% (95% CI: 0.21%, 0.44%) percent increase in the hospitalization rate per ppb increase in warm-season O_3_. In areas with even lower warm-season O_3_ levels, specifically below 20 ppb, the percent changes in hospitalization rates for stroke, HF, and AF per ppb increase in warm-season O_3_ were 0.79% (95% CI: 0.58%, 1.01%), 0.70% (95% CI: 0.46%, 0.95%), 0.71% (95% CI: 0.40%, 1.02%), respectively.

**Figure 2:**
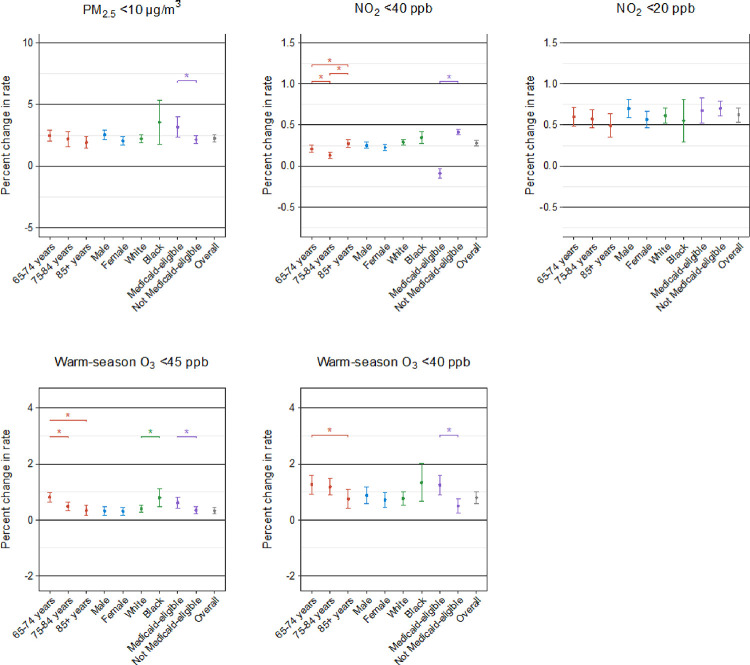
Percent change in hospitalization rate for stroke associated with 1-μg/m^3^ increase in long-term exposure to PM_2.5_ and 1 ppb increase in long-term exposure to NO_2_ and warm-season O_3_ at low concentrations in stratified analyses by age, sex, race, and Medicaid eligibility in three-pollutant double negative control models. Note: * indicates statistically significant differences (*P*<0.05).

**Figure 3:**
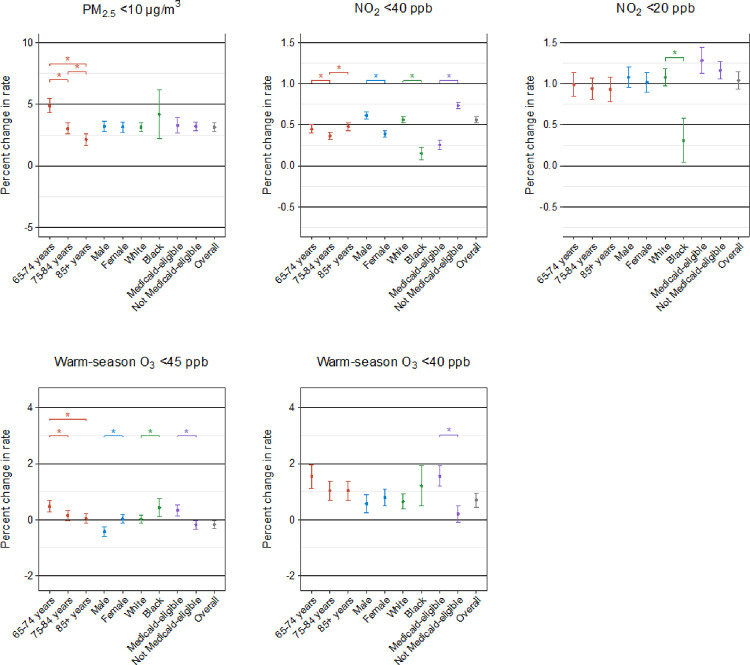
Percent change in hospitalization rate for heart failure associated with 1-μg/m^3^ increase in long-term exposure to PM_2.5_ and 1 ppb increase in long-term exposure to NO_2_ and warm-season O_3_ at low concentrations in stratified analyses by age, sex, race, and Medicaid eligibility in three-pollutant models using double negative control adjustment. Note: * indicates statistically significant differences (*P*<0.05).

**Figure 4:**
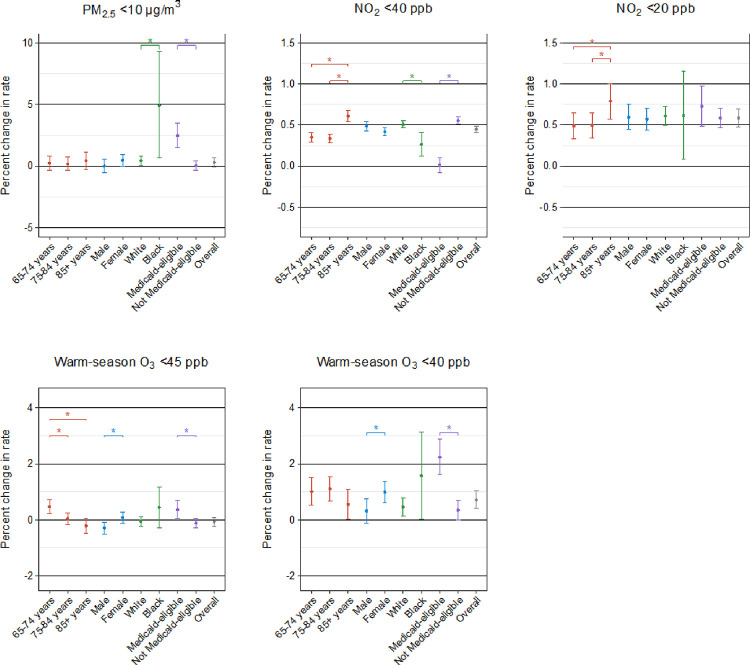
Percent change in hospitalization rate for atrial fibrillation and flutter associated with 1-μg/m^3^ increase in long-term exposure to PM_2.5_ and 1-ppb increase in long-term exposure to NO_2_ and warm-season O_3_ at low concentrations in stratified analyses by age, sex, race, and Medicaid eligibility in three-pollutant models using double negative control adjustment. Note: * indicates statistically significant differences (*P*<0.05).

We conducted stratified analyses by individual demographic characteristics to identify the subgroups vulnerable to the harmful effects of PM_2.5_, NO_2_, and warm-season O_3_. The results of the stratified analyses for stroke, HF, and AF from three-pollutant models are shown in Figs. 2, 3, and 4, respectively. We found that the observed positive associations in the overall analyses generally persisted in demographic subgroups. In general, the patterns of the potential effect modification by demographics were similar in models with and without adjustment for co-pollutants, despite some changes in the magnitude and statistical significance of the subgroup-specific effect estimates (**Supplementary Figs. 1, 2, and 3**). All the detailed numeric results of the stratified analyses are presented in **Supplementary Tables 4, 5, and 6.**

In the association of long-term PM_2.5_ exposure with stroke and AF, we identified Medicaid eligibility as a significant modifier, with a higher risk seen in individuals who were eligible for Medicaid than those who were not. We also found a larger effect of PM_2.5_ on all three outcomes for black people compared to white people, although the difference did not reach statistical significance for stroke and AF. In addition, age modified the PM_2.5_ association for HF with a stronger effect in the younger group (aged 64–75 years), but this modification pattern was not observed for stroke or AF. In contrast, we found no evidence of any effect modification by sex on the association of all outcomes in relation to PM_2.5_.

For long-term exposure to NO_2_ below 40 ppb, individuals aged over 84 years and those who were not Medicaid-eligible were at greater risk of stroke. We observed similar effect modification patterns by age and Medicaid eligibility in the associations of HF and AF with NO_2_. Regarding the modification by sex, males were at greater NO_2_-associated risk of HF compared to females. At the same time, white people exhibited a significantly higher NO_2_-associated risk of HF and AF compared to black people. However, the modification analyses for NO_2_ below 20 ppb were not apparent, with only stronger estimates observed for white individuals in relation to HF and for the oldest age group in relation to AF.

In terms of long-term exposure to warm-season O_3_ below 45 ppb, individuals aged 64–75 years, black individuals, and Medicaid-eligible individuals were found to be more susceptible to stroke and HF. Additionally, we observed positive associations between warm-season O_3_ and AF for individuals aged 65–74 years and those eligible for Medicaid, whereas other subgroups showed non-significant associations. For warm-season O_3_ below 40 ppb, we saw larger effects among Medicaid-eligible individuals across all outcomes. Furthermore, females were at greater risk of AF due to exposure to warm-season O_3_.

## 4. Discussion

Among US Medicare participants, we found that long-term exposure to low-level PM_2.5_ (< 10 μg/m^3^), NO_2_ (< 40 or 20 ppb), and warm-season O_3_ (< 45 or 40 ppb) could significantly increase the rate of hospitalizations for stroke in three-pollutant models that accounted for correlations between co-existing air pollutants and controlled for unmeasured confounders using negative controls. We also observed positive associations between PM_2.5_ and NO_2_ with HF and AF, although the effect of PM_2.5_ on AF was non-significant. When applying a more restrictive threshold to NO_2_ and warm-season O_3_, the estimates became even stronger. Black people and Medicaid-eligible people appeared to be more vulnerable to the risk attributable to PM_2.5_ and warm-season O_3_. For the NO_2_-related risk, very elderly people and those who were not Medicare-eligible may be more susceptible to all outcomes, and white people may be more susceptible to HF and AF. We designed a pair of negative control exposure and outcome variables to capture any uncontrolled confounding. If the assumption of linearity between unmeasured covariates with exposure and negative exposure control holds, the double negative control adjustment can strengthen the causal interpretation of our observed associations. The GLM method yielded comparable results with the double negative control approach, exhibiting only slight differences in the effect size estimates. Such discrepancies may be attributable to unadjusted confounding bias. The consistent findings derived from these two statistical methods demonstrate the robustness of our results to different model adjustments, suggesting that any omitted confounding bias is small, and, in the case of PM_2.5_ and NO_2_, negative. A previous study reported that greater control for SES resulted in increased effect sizes for PM_2.5_^43^.

Our study has a special emphasis on long-term exposure to low-level air pollution below the annual US EPA limits. While a growing number of prior studies have revealed increased health risks at lower levels of air pollution exposure under regulatory standards, most have focused on all-cause and cardiovascular mortality ^11,12,44,45^. However, the available evidence concerning cardiovascular disease risk at these lower pollution levels remains limited. For instance, in a large population-based Canadian cohort, Bai et al. ^7^ found the concentration-response curves for congestive HF with long-term exposure to PM_2.5_ and NO_2_ to be supralinear with no discernable threshold values. They also observed a sublinear relationship for O_3_ with an indicative threshold. Similarly, Brunekreef et al. ^15^ observed steeper slopes at low exposures to PM_2.5_ below 15 μg/m^3^ and NO_2_ below 40 μg/m^3^ in the supralinear associations for stroke incidence, based on data from 22 European cohorts in the European Study of Cohorts for Air Pollution Effects Project. Several previous studies of the Medicare population have found a greater risk of a range of cardiovascular outcomes when restricted to lower exposures ^9,13,14,27^. Our main finding adds to epidemiologic evidence of potential population-level health concerns at pollution levels conventionally considered safe and provides some assurance that the associations are not biased by unmeasured confounding. More importantly, this highlights the need to reassess the current air quality guidelines and tighten pollution control policies and measures.

This study also supplemented the limited epidemiologic evidence regarding the long-term effects of multiple air pollutants on cause-specific cardiovascular morbidity. We concluded that long-term exposure to PM_2.5_, NO_2_, and warm-season O_3_ even at low concentrations could be associated with an increase in the rate of hospitalizations for major cardiovascular diseases. The adverse association was more pronounced for stroke and HF than for AF. Our findings are in accordance with some of the existing literature. Prior studies of the Medicare population using diverse methodologies and different ranges of exposure have reported significant positive associations of all our studied outcomes with PM_2.5_, NO_2_, and warm-season O_3_^13,31^. A review and meta-analysis identified five studies of long-term exposure to PM_2.5_ and stroke incidence from North America and Europe and found a 6.4% (95% CI: 2.1%, 10.9%) increase in the hazard for each 5-μg/m^3^ increase in PM_2.5_^46^. A more recent review article reported that each 10-μg/m^3^ increase in long-term PM_2.5_ exposure could be associated with an increased risk of 13% (95% CI: 11%, 15%) for incident stroke, synthesizing the results of fourteen studies across the globe ^47^. In a large population-based study of about 5.1 million adults living in Ontario, Canada, annual PM_2.5_, NO_2_, and O_3_ were found to elevate the risk of HF with HRs of 1.05 (95% CI: 1.04, 1.05), 1.02 (95% CI: 1.01, 1.04), and (95% CI: 1.02, 1.03) per each interquartile range increase in exposure, respectively. Similarly, a prospective study in the UK reported positive associations of incident HF with long-term PM_2.5_ and NO_2_^8^. Yue et al. ^10^ conducted a systematic review and meta-analysis to quantify the association between air pollutants and AF based on eighteen studies. They indicated that exposure to all air pollutants including PM_2.5_ and NO_2_ had a deleterious impact on AF onset in the general population. By contrast, several other studies reported null relationships between air pollution and the risk of these outcomes ^48–51,51^. It is worth noting that direct comparisons across these studies might be challenging because of potentially heterogeneous air pollution ranges and diverse demographic characteristics of study populations.

Multiple pathophysiological mechanisms have been proposed to explain the detrimental cardiovascular effects of air pollution. It is widely accepted that air pollution can trigger systemic inflammation, oxidative stress reactions, and dysfunction of the autonomic nervous system ^1^. The autonomic imbalance can further result in increases in cardiac frequency and arterial pressure, and a reduction in heart rate variability ^52^. Numerous experimental studies have demonstrated that these responses may further instigate endothelial dysfunction, atherosclerosis, and vascular dysfunction ^52,53^. Another plausible mechanism underlying the onset of cardiovascular diseases is that inhaled irritants can traverse the pulmonary epithelium and directly enter the blood circulation and cardiac organs, which may alter blood coagulability and contribute to thrombus formation ^54^. The heart failure hospitalization was the most vulnerable outcome possibly because it was the common consequence of most cardiovascular diseases, especially for elderly people.

Environmental justice is an increasing concern and we found evidence that independent of differences in exposure, some disadvantaged groups had worse responses to any given level of air pollution. Specifically, we identified Medicaid eligibility as a positive modifier of the association of low-level PM_2.5_ and warm-season O_3_ with both stroke and AF. This suggests a greater vulnerability for lower-SES individuals even when residing in low-pollution regions, as Medicaid coverage is provided for low-income elderly beneficiaries to expand their healthcare access ^55^. Low SES has been determined as a significant risk factor for cardiovascular diseases because socio-economically disadvantaged individuals tend to have poorer health, higher psychosocial stress, and a propensity for unhealthy behaviors and lifestyles ^56^. In addition to Medicaid eligibility, we found that the effect sizes for effects of PM_2.5_ and warm-season O_3_ on all outcomes were more pronounced for Black individuals compared to white individuals. The tendency of a higher susceptibility among Blacks is consistent with much of the existing evidence ^13,57^. Black populations have been disproportionately affected by the detrimental health impacts of historic discrimination and ongoing racial segregation, and this study demonstrates additional susceptibility to air pollution. Additionally, while we observed increased susceptibility to warm-season O_3_ in individuals aged 65–74 years, the specific underlying reasons for this pattern remain unclear. It is likely that a lower baseline risk in this age group may influence these findings.

In terms of the adverse effects of NO_2_, our results indicated that people aged ≥ 85 years, males, white people, and those who were not Medicaid-eligible may be more vulnerable to at least one cardiovascular disease we studied. First, an increased risk in the oldest group is understandable, given that advanced age significantly drives the deterioration of cardiovascular functionality in older people ^58^. Relative to age differences, sex as a potential modifier of cardiovascular risk in relation to air pollution as well as the relevant biological mechanisms has been more underappreciated. While some researchers found a more prominent NO_2_-attributed cardiovascular risk among males ^59,60^, which is comparable to our finding for HF, there is no consensus on this question ^32,61^. Our findings of a higher susceptibility among the very elderly and males are not conclusive, but we think that paying more attention to these questions can be meaningful to inform more scientific appointments of preventive medical care in the future. Interestingly, when we looked at the modification by race and Medicaid eligibility, the greater susceptibility for NO_2_ seen in white individuals and non-Medicaid eligible individuals contrasts with our findings for PM_2.5_. Such inconsistent results in the modifying roles of demographics and SES exist in the literature examining the association between air pollution and cardiovascular health, which may have to do with different air pollutants, specific outcomes, and neighborhood samples ^9,62,63^. In fact, the specious modification patterns we found for NO_2_ are unlikely but still possible. As a pollutant predominantly coming from urban origins and often transported on a local scale, NO_2_ can vary by urbanicity level ^64^. It is reasonable to assume that NO_2_ might be more of a proxy for commercial activities, since its emissions from other major sources (e.g., diesel traffic, fuel combustion, power plants) have been reduced in recent years ^65,66^. Therefore, the observed higher vulnerability in white and Medicaid-eligible individuals might be partially accounted for by their higher access to urbanization or commercial activities. In addition, we should also note that our estimate is a measure relative to the baseline risk and does not necessarily represent the magnitude of its absolute attributable risk. For example, the lower baseline risk of HF hospitalization rate in white beneficiaries might have exaggerated the magnitude of relative risk.

Our study has multiple strengths. Foremost is the use of a double negative control approach. This methodology provides an alternative tool to instrumental variables to control for omitted confounding and thus enhance the credibility of the estimated associations. We also thoroughly considered a variety of cardiovascular risk factors to reinforce the confounding adjustment. Another notable strength is that we leveraged the data from the Medicare population. The data that we used was from a very large nationwide cohort, which ensured sufficient statistical power and increased the generalizability of our results to the population that suffers over three quarters of the deaths in the US. Furthermore, the exposure data were derived from high-quality models with a fine resolution and satisfactory predictive accuracy, further assuring the reliability of our analyses. Moreover, compared to restricting the analyses to low exposures in ZIP code-year combinations in prior Medicare studies ^13,67^, the selection criteria applied in this study are somewhat more rigorous by imposing low-exposure constraints over the 17-year study duration. Hence, the possibility of mistakenly including the individuals impacted by past higher exposures was reduced. Last, we attempted to address the correlations among air pollutants and more accurately estimate the independent effect of each exposure by constructing both single- and three-pollutant models.

Some limitations of this study should also be cautioned. First, we may not generalize the conclusions to younger populations or highly polluted regions. Second, there could be residual or unmeasured confounding because the assumptions for the double negative control method might be violated. However, we considered a series of major confounders, ranging from possible meteorological conditions, and health behavioral factors, to socioeconomic measures, which should have captured most of the confounding associations. It is noteworthy that we controlled for co-exposures of other air pollutants using the three-pollutant models as well. Admittedly, the moderate correlation between annual PM_2.5_ and NO_2_ concentrations may indicate potential collinearity and the risk of over-controlling issues. Third, the ZIP code-level air pollution data derived from exposure models may not fully represent true personal exposures. Specifically, our exposure metrics did not account for the exposures occurring distant from the participants’ residences. However, the National Human Activity Pattern Survey reported that US adults spent 69% of their time at home and 8% of the time immediately outside their home ^68^. Older people may spend even more time at home, implying that the exposure misclassification would be relatively minor. Another concern is that the variations in personal exposures caused by different indoor activity patterns and building features might not be captured by the neighborhood metrics. Nevertheless, the resulting error is likely a Berksonian exposure error and may cause little bias ^69^. Some residual prediction errors of exposure models may be present, but they should be minimal because we studied low air pollutant concentrations. Last, we accessed hospital discharge diagnoses from the administrative Medicare database as the morbidity measure, which may not capture some cases with milder symptoms. However, since the potential outcome classification is not expected to relate to air pollution, it will introduce a non-differential bias towards the null.

## 5. Conclusions

Using a double negative control approach, we found positive associations of long-term exposure to PM_2.5_, NO_2_, warm-season O_3_ at low concentrations with the hospitalization rate of stroke, HF, and AF in US Medicare older adults. Black and Medicaid-eligible people may be more susceptible to the risk attributed to PM_2.5_ and warm-season O_3_, whereas those who are very elderly, white, and non-Medicaid-eligible may be at greater risk attributed to NO_2_. Our findings suggest that the current NAAQS for annual PM_2.5_ and NO_2_ may not be adequate to minimize the cardiovascular disease burden. Future guidelines for warm-season O_3_ could be warranted.

## Figures and Tables

**Table 1:** Summary of ZIP code-level air pollution, meteorological covariates, and SES covariates in the low pollution areas from 2000 through 2016.

Covariates	PM_2.5_ <10 μg/m^3^	NO_2_ < 40 ppb	NO_2_ < 20 ppb
N (ZIP code)	5,848	26,583	12,281
N (beneficiaries)	12,667,627	63,657,996	212,454,83
**Air pollution concentration**			
PM_2.5_ (μg/m^3^)	5.9 (1.8)	9.6 (3.0)	9.3 (2.8)
NO_2_ (ppb)	12.8 (7.6)	14.7 (7.1)	9.9 (3.5)
Warm-season O_3_ (ppb)	44.6 (7.3)	45.0 (5.4)	44.5 (4.6)
**Meteorological covariates**			
Summer average temperature (°C)	17.6 (4.3)	20.2 (3.7)	20.2 (3.8)
Winter average temperature (°C)	3.8 (6.8)	6.2 (5.8)	5.6 (5.9)
Summer average RH (%)	58.8 (14.4)	66.7 (9.4)	67.8 (7.8)
Winter average RH (%)	66.8 (10.4)	66.9 (7.1)	67.4 (6.3)
**SES covariates**			
Percent Black (%)	1.8 (5.4)	8.5 (16)	7.2 (14.7)
Percent Hispanic (%)	10.3 (16.2)	7.8 (14.4)	4.9 (10.8)
Median household income ($)	47,911 (17,976)	48,420 (20,205)	43,105 (14,352)
Median house value ($)	173,484 (135,217)	149,977 (124,612)	117,697 (89,262)
Percent owner occupied (%)	75.5 (11.6)	74.2 (14.0)	77.9 (9.7)
Percent education < high school (%)	22.2 (14.3)	28.1 (16.1)	30.6 (16.1)
Population density (persons/mi^2^)	435 (1,684)	841 (2,095)	144 (446)
Percent ≥ 65 below poverty (%)	9.3 (7.4)	10.1 (7.7)	11.2 (7.6)
Percent annual HbA1c test (%)	82.8 (8.6)	83.5 (5.9)	83.8 (6.1)
Percent ambulatory visit (%)	78.3 (7.4)	79.8 (6.0)	80.9 (6.5)
Percent eye exam (%)	69.2 (7.5)	67.3 (6.7)	67.5 (7.5)
Percent LDL test (%)	76.7 (9.9)	78.6 (7.3)	78.1 (7.6)
Percent mammogram (%)	65.2 (8.7)	64.1 (7.3)	64.3 (8.0)
Distance to nearest hospital (km)	16.3 (14.9)	12.2 (10.6)	15.4 (10.8)
Lung cancer rate (‰)	0.4 (4.8)	0.4 (2.2)	0.5 (2.2)
Ever smokers (%)	47.8 (7.6)	47.3 (7.4)	47.9 (7.9)
Mean BMI (kg/m^2^)	28.1 (3.2)	28.1 (2.5)	28.7 (3.0)
**Covariates**	**Warm-season O_3_ < 45 ppb**	**Warm-season O_3_ < 40 ppb**	
N (ZIP code)	3,740	1,120	
N (beneficiaries)	12,995,867	5,262,809	
**Air pollution concentration**			
PM_2.5_ (μg/m^3^)	7.8 (2.8)	7.9 (2.5)	
NO_2_ (ppb)	15.8 (9.4)	17.2 (7.9)	
Warm-season O_3_ (ppb)	37.3 (3.9)	33.2 (2.9)	
**Meteorological covariates**			
Summer average temperature (°C)	19.1 (5.1)	20.8 (5.7)	
Winter average temperature (°C)	7.6 (8.7)	13.2 (7.5)	
Summer average RH (%)	68.9 (7.1)	70.4 (6.7)	
Winter average RH (%)	70.0 (7.2)	71.5 (6.7)	
**SES covariates**			
Percent Black (%)	6.5 (13.8)	7.8 (13.8)	
Percent Hispanic (%)	14.8 (22.2)	24.8 (28.0)	
Median household income ($)	52,236 (23,004)	55,294 (26,806)	
Median house value ($)	230,706 (191,275)	289,056 (239,879)	
Percent owner occupied (%)	69.1 (18.5)	64.2 (18.0)	
Percent education < high school (%)	24.4 (16.4)	25.8 (19.3)	
Population density (persons/mi^2^)	3,446 (10,154)	3,874 (5,701)	
Percent ≥ 65 below poverty (%)	10.3 (8.4)	11.8 (10.1)	
Percent annual HbA1c test (%)	84.6 (4.8)	83.7 (3.8)	
Percent ambulatory visit (%)	76.6 (7.2)	75.6 (5.8)	
Percent eye exam (%)	70.5 (6.3)	69.3 (4.9)	
Percent LDL test (%)	80.6 (5.5)	81.8 (4.9)	
Percent mammogram (%)	66.1 (7.3)	63.9 (6.9)	
Distance to nearest hospital (km)	10.2 (10.6)	7.2 (9.5)	
Lung cancer rate (‰)	0.4 (6.0)	0.4 (7.6)	
Ever smokers (%)	47.6 (7.7)	45.0 (7.9)	
Mean BMI (kg/m^2^)	27.4 (1.7)	27.1 (1.4)	

Note: Numbers in the table are presented as Mean (SD) for ZIP code-level covariates.

## Data Availability

The authors do not have permission to share Medicare data but interested investigators can obtain it by applying for their own Data Use Agreement to the Center for Medicare and Medicaid Services. The air pollution data is freely available online at the NASA SEDAC website (https://sedac.ciesin.columbia.edu/data/collection/aqdh/sets/browse).
